# Cardiac Monitoring with Textile Capacitive Electrodes in Driving Applications: Characterization of Signal Quality and RR Duration Accuracy

**DOI:** 10.3390/s25196097

**Published:** 2025-10-03

**Authors:** James Elber Duverger, Geordi-Gabriel Renaud Dumoulin, Victor Bellemin, Patricia Forcier, Justine Decaens, Ghyslain Gagnon, Alireza Saidi

**Affiliations:** 1 Institut de Recherche Robert-Sauvé en Santé et en Sécurité du Travail, Montréal, QC H3A 3C2, Canada; james.elber-duverger@irsst.qc.ca; 2 Department of Electrical Engineering, École de Technologie Supérieure, Université du Québec, Montréal, QC H3C 1K3, Canada; geordi-gabriel.renaud-dumoulin.1@ens.etsmtl.ca (G.-G.R.D.); victor.bellemin.1@ens.etsmtl.ca (V.B.); ghyslain.gagnon@etsmtl.ca (G.G.); 3 CTT Group, Saint-Hyacinthe, QC J2S 1H9, Canada; pforcier@gcttg.com (P.F.); jdecaens@gcttg.com (J.D.)

**Keywords:** cardiac monitoring, ECG sensor, textile capacitive electrode, automobile, automotive application, driving application, capacitive sensing, ECG signal, signal quality index, driver health status

## Abstract

Capacitive ECG sensors in automobiles enable unobtrusive heart rate monitoring as an indicator of a driver’s alertness and health. This paper introduces a capacitive sensor with textile electrodes and provides insights into signal quality and RR duration accuracy. Electrodes of various shapes, sizes, and fabrics were integrated at various positions into the seat back of a driving simulator car seat. Seven subjects completed identical driving circuits with their cardiac signals being recorded simultaneously with textile electrodes and reference Ag-AgCl electrodes. Capacitive ECG signals with observable R peaks (after filtering) could be captured with almost all pairs of textile electrodes, independently of design or placement. Signal quality from textile electrodes was consistently lower compared with reference Ag-AgCl electrodes. Proximity to the heart or even contact with the body seems to be key but not enough to improve signal quality. However, accurate measurement of RR durations was mostly independent of signal quality since 90% of all RR durations measured on capacitive ECG signals had a percentage error below 5% compared to reference ECG signals. Accuracy was actually algorithm-dependent, where a classic Pan–Tompkins-based algorithm was interestingly outperformed by an in-house frequency-domain algorithm.

## 1. Introduction

### 1.1. Background and Motivation

The progress in contactless vital signs monitoring is increasingly transforming driver safety by enabling real-time assessment of health and alertness. For instance, various studies have shown that a decrease in heart rate variability (HRV), particularly in specific frequency bands, is correlated with increased fatigue and drowsiness in drivers. This is because HRV reflects the dynamic balance between the sympathetic and parasympathetic nervous systems, thus providing direct insight into the physiological processes that regulate alertness and safe driving ability [1,2,3]. Monitoring such physiological parameters in a vehicle environment suggests the potential for effective, real-time solutions to detect and prevent driver drowsiness [4]. Indeed, drowsiness and fatigue while driving are major factors contributing to serious and fatal accidents worldwide. In the United States, they are involved in approximately 21% of fatal accidents, causing more than 6400 deaths annually. These figures are very similar to road accident statistics in Canada. In Europe and in Asia, driver drowsiness and reduced vigilance are responsible for at least one-third of fatal accidents on highways [5,6,7,8].

Traditional galvanic ECG sensors have significant limitations for monitoring drivers in real-world conditions. Their operation relies on direct skin contact, making them susceptible to movement and variations in electrode–skin contact. This constraint also leads to discomfort during prolonged use and often requires adjustments or the use of intrusive devices to maintain contact. Although effective in the laboratory, these sensors lose reliability in a vehicle, where driving movements generate interference that limits their practical use [9,10]. Consequently, the demand is growing for integrated, non-intrusive systems to ensure a better balance between comfort, robustness, and reliability [9,11,12].

Some early automotive research explored placing galvanic ECG electrodes on the steering wheel to monitor heart rate [13,14,15,16,17,18,19,20]. While feasible, this method requires both hands to maintain contact with separate conductive areas, making it susceptible to common driving behaviors such as one-handed steering or frequent grip changes [9]. To overcome these limitations, a hybrid detection approach has been proposed that combines a steering wheel with a capacitive electrode integrated into the driver’s seat, thus accommodating natural driving habits [21,22,23].

### 1.2. Capacitive ECG Technology and Use Cases

Capacitive coupling ECG (cECG) provides an alternative to the traditional galvanic ECG method, using high input impedance electrodes capable of monitoring the heart’s electrical activity through clothing. In such a system, a capacitor is created with the body skin and a conductive element is integrated into the car seat or backrest, with the clothing acting as the insulating dielectric layer between the electrodes [24,25]. Based on the creation of such a capacitor, the electrical signals in the body associated with heart activity modulate the local electromagnetic fields in the body, which will be coupled to the displacement current of the capacitor. Such an interaction allows the heart signal to be detected [24]. As the collected cECG signals are usually smaller than with galvanic electrodes, a low-noise operational amplifier is frequently associated with each capacitive electrode. In such a system, the compensation of the common mode is usually insured by an active driven ground [9].

Pioneer studies regarding the integration of cECG systems were dedicated to the digital health domain and daily life objects to promote remote health monitoring. These works included beds [26,27,28,29], toilet seats [30], wearable setups [31], and smart seats [32,33,34,35]. Although various cECG systems have continued to advance [36,37], the most valuable insights for optimizing integration into vehicle seats come from progress in smart seat designs equipped with cECG [24,38,39,40,41], given their more similar configuration. Indeed, cECG monitoring was only introduced to the automotive sector in the late 2000s [21,42,43], and since then the scientific community has been interested in integrating this technology into automotive seats, with studies focusing on electronic design, as well as the type, material, shape, number, and location of capacitive electrodes [9,25,44].

### 1.3. Circuit Architectures for cECG

Regarding the electronic design for cECG systems for both regular and vehicle seats, many studies incorporated improvement into the basic circuitry, notably, a bias resistor to discharge static electricity and stabilize the high-pass filter for optimal signal transfer [42,45], an impedance matching circuit to balance high impedance between electrodes and skin [43], and an additional capacitor in series to minimize the overall capacitance change, and hence decrease ECG distortion due to impedance mismatch between a pair of electrodes [42]. Some others explored the inclusion of a driven ground plane circuit to minimize common mode voltage interference from environmental sources [25,45,46]. In that case, the steering wheel can be used as a low impedance reinjection pathway of the common mode interference signal [22]. Additionally, negative feedback techniques or driven-right-leg (DRL) circuits have been employed to improve system stability and address challenges associated with electrode inhomogeneity, such as variations in clothing or electrode coverage [24,44,45].

### 1.4. cECG Electrode Materials and Shapes

Many studies on integrating cECG into regular seats [32,35,39,40] or vehicle seats [21,25,44,45,46,47,48] have opted for metallic capacitive electrodes, even though they may present challenges for seat integration and user comfort. By contrast, conductive textiles offer a conformable and comfortable alternative for capacitive electrode design [24], while also enabling direct and seamless integration into seat covers [42,49,50].

Textile electrodes for cECG systems were most commonly developed with woven conductive materials for conventional seats [38,41] and vehicle seats applications [42,43,49,50]. Only a few studies have explored electrodes based on knitted conductive fabric [24]. Some of the studies on seat-integrated cECG systems have also examined the influence of electrode geometry on signal quality, either by exploring the impact of the size of rectangular electrodes manufactured with knitted fabric [24], or by comparing the influence of square or circular textile capacitive electrodes of different sizes [42].

### 1.5. Number of Electrodes and Spatial Positioning

Moreover, a significant proportion of seat-integrated systems in the literature consist of two capacitive electrodes. Some of these electrodes were placed in the lumbar region [40,49,50] or the thoracic area [21,32,33,35,39,43], with certain studies including reliability tests on the road [21,49,50] or on a simulator [43]. A few others explored the integration of couple electrodes into a vehicle seat, according to skew electrode arrangements [45], based on averaged pressure data from diverse subjects [47], or in such a way as to avoid interference from existing devices like heaters and ventilators [42].

Notably, even in multielectrode setups, signals were usually captured from just one pair of electrodes at a time, with very few exceptions [51]. For an aircraft pilot’s seat with integrated textile electrodes, a Signal-to-Noise Ratio (SNR) optimization algorithm was used to select the optimal pair of electrodes from eight fixed electrodes, to record signals from four subjects under static conditions [24]. For a vehicle seat with six integrated stainless-steel electrodes, R-peak amplitude and noise analysis were used to identify the optimal cECG channel for signal recording during driving simulations with ten subjects [25,46], while a separate study compared the performances of the three channels of the same system during driving simulations with twenty subjects [44].

To our knowledge, very few studies have focused on exploring versatile electrode placement. Within this framework, the optimal positioning of a pair of copper electrodes on a vehicle seat has been explored through nine symmetrical and asymmetrical electrode arrangements by analyzing signal amplitude and noise effects in data collected from two male subjects during two-hour driving tests [48]. Finally, the placement of two textile electrodes on a regular seat was investigated through six symmetrical and asymmetrical positions by comparing the SNR calculated for each test performed on one subject under static conditions [41]. Indeed, over the past ten years, research has focused primarily on fixed placement, with flexible electrode placement remaining largely unexplored.

### 1.6. Unexplored Research Questions

While these studies provide valuable insights, the small sample size and limited participant diversity both limit the extent to which the findings can be generalized. Also, previous studies seem to explore only a few parameters of the cECG system at a time. Overall, no article has presented practical and realistic insight to the following question: where and how should textile electrodes be integrated into a car seat to obtain good quality cECGs in the context of monitoring vital signs while driving? Our study aims to fill this gap in the literature in the following ways:

1. Design textile electrodes using industrial embroidering methods, a realistic and seamless approach in the context of seat cover design in the automotive industry.
2. Integrate pairs of electrodes into the seat back of a car seat in a practical and user-friendly way, as expected for the normal use of a vehicle.
3. Integrate the electrodes following very diverse configurations to cover a wide range of situations in terms of placement and form factor.
4. For data collection, recruit numerous subjects of varying body sizes, to obtain generalizable results.
5. Evaluate the performance of the electrode integration based on known or expected needs in the context of monitoring vital signs while driving, notably noise level and RR duration accuracy.

## 2. Materials and Methods

### 2.1. Capacitive Sensing: Principle and Implementation

#### 2.1.1. Design and Prototyping of Textile Electrodes

**Figure 1:**
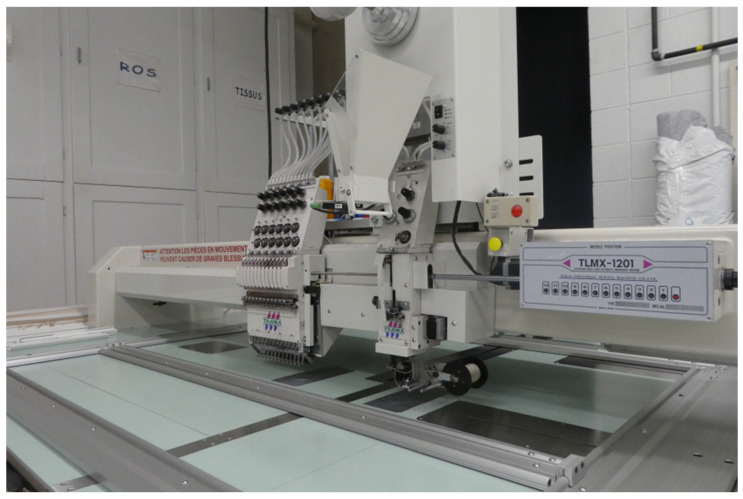
Tajima TMLX-1201 embroidery machine.

A conventional embroidery process was used to design the electrodes, utilizing a Tajima TMLX-1201 (from Tajima Industries Ltd., Kasugai, Japan) embroidery machine with a regular head (Figure 1). The conductive yarn used was the silver-plated polyamide 66 Yarn 235/34 dtex 2-ply (from Statex Produktions- und Vertriebs GmbH, Bremen, Germany) composed of 99% pure silver-plated polyamide yarn 560/68 dtex with anti-tarnished coating, forecasting a linear electrical resistance < 100 Ω/M and mechanical tenacity of 46 cN/tex. The conductive yarn was embroidered on a black substrate made of 65% polyester/35% cotton, WR (water repellent) poplin fabric of 180 g/m^2^ surface mass density. Yarn tension was carefully adjusted to ensure both high stitch quality and reliable electrical continuity of the conductive patterns. On the embroidery machine, bobbin and needle tensions were balanced so that the conductive needle thread was slightly drawn beneath the substrate, in accordance with embroidery quality standards. Given the conductive yarn friction, the needle tension was set to the maximum allowable level (T = 5 on the machine scale) without causing breakage. This setting maintained the stability and continuity of the embroidered conductive patterns while preventing thread slack and substrate deformation. We adopted a single-layer embroidery approach, relying on intact conductive yarns throughout the embroidery process. Conductivity was further ensured by inter-thread contact between adjacent yarns. Electrical performance was ensured by adjusting stitch density rather than layering, especially as additional layers increase the risk of needle-induced damage to previously embroidered yarns. Structural stability was provided by the woven substrate, offering sufficient resistance to stretching and deformation without the addition of volumetric materials such as 3D puff or filler yarns.

Three types of embroidered electrodes were prototyped using a medium embroidery density:

- A.73.41 with a surface area of 7.62 cm × 7.62 cm (58.06 cm^2^);
- A.73.42 with a surface area of 11.43 cm × 5.08 cm (58.06 cm^2^);
- A.73.50 with a surface area of 5.08 cm × 5.08 cm (25.81 cm^2^).

**Figure 2:**
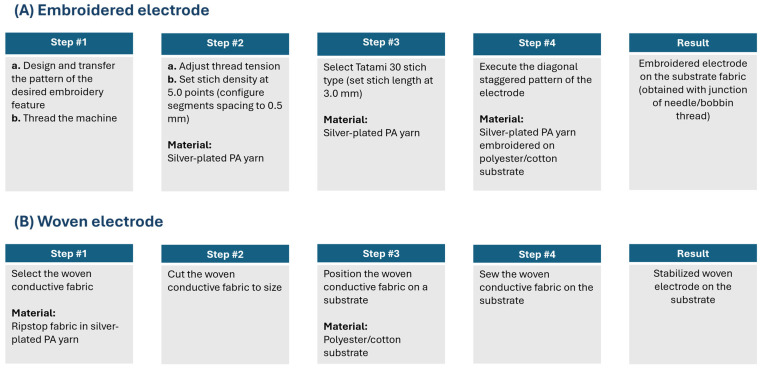
Prototyping process of the electrodes. (**A**) Embroidered electrode. (**B**) Woven electrode.

The prototyping process of the electrodes is illustrated in Figure 2. To create embroidered electrodes, we used a stitch density of 5.0 points, corresponding to a spacing of 0.5 mm between the embroidery segments, to ensure a proper balance between fabric coverage and flexibility. The filling was performed using the Tatami 30 stitch type, characterized by a stitch length of 3.0 mm. This type of filling creates a diagonal visual effect due to the staggered arrangement of stitches, resulting from the junction of the needle thread and the bobbin thread.

A woven electrode was also prototyped for comparison purposes:

- A.73.46 with a surface area of 7.62 cm × 7.62 cm (58.06 cm^2^).

The woven electrode was prepared with PF152RS/P, a conductive fabric by Shieldex (from Statex Produktions- und Vertriebs GmbH, Bremen, Germany), manufactured in a RipStop structure weave from silver-plated polyamide yarn, and providing a surface electrical resistance (sheet resistance) < 50 Ω/sq. This woven conductive fabric was then sewn onto the same type of substrate used for the embroidered electrode to provide mechanical stability. The substrate of both embroidered and woven electrodes contained a VELCRO^®^ (by Velcro USA Inc., Manchester, NH, USA) system in its contour, enabling it to be integrated into the surface of the seat back.

**Figure 3:**
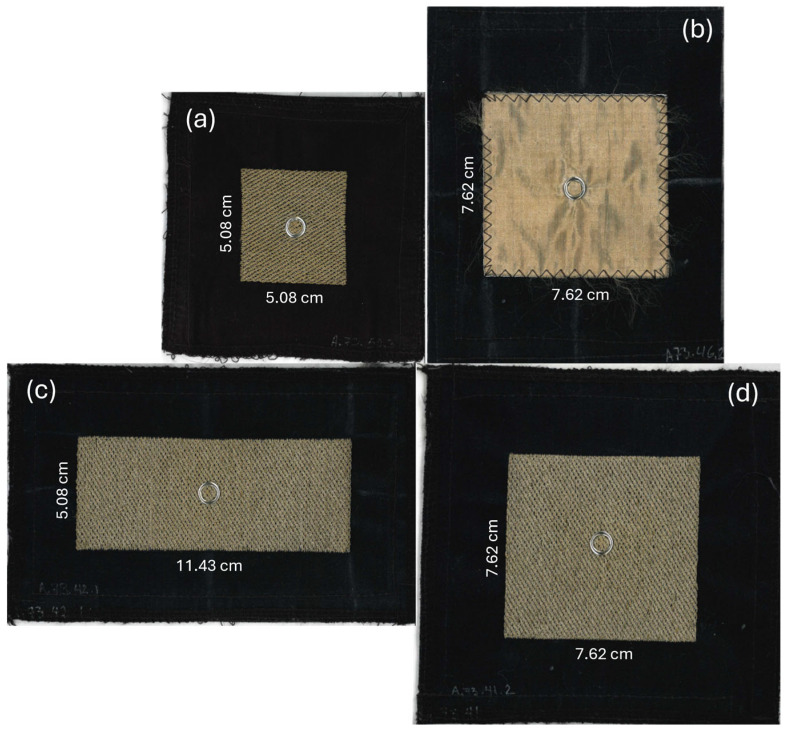
Textile capacitive electrode samples. (**a**) Electrode A.73.50: embroidered, square, 25.81 cm^2^. (**b**) Electrode A.73.46: woven, square, 58.06 cm^2^. (**c**) Electrode A.73.42: embroidered, rectangle, 58.06 cm^2^. (**d**) Electrode A.73.41: embroidered, square, 58.06 cm^2^. The gray metal ring seen in the center of each electrode is the socket side of a snap-in connector that allows the cECG active circuit board to be attached directly to the back of the electrode.

Figure 3 shows samples of all the electrodes used in the study. The gray metal ring seen in the center of each electrode is the socket side of a snap-in connector that allows the cECG active circuit board to be attached directly to the back of the electrode.

#### 2.1.2. ECG Acquisition Data Pipeline: Hardware and Software

**Figure 4:**
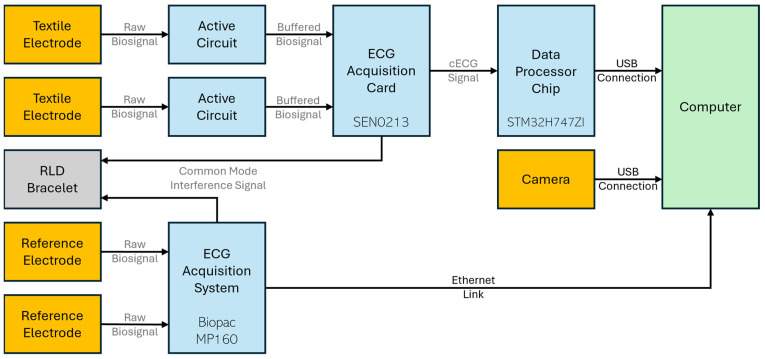
ECG acquisition pipeline.

The ECG acquisition data pipeline is illustrated in the conceptual diagram of Figure 4. The pipeline has five inputs, in orange: (a) two textile capacitive electrodes integrated into the car seat, (b) two Ag-AgCl reference electrodes (Red Dot™ Foam Monitoring Electrode 2560, 3M Company, Saint-Paul, MN, USA) attached to the subject’s torso, and (c) a Webcam PC camera (model ELP-USBFHD05MT-KL170IR, Shenzhen Ailipu Technology Co Ltd., Shenzhen, China) recording the subject’s driving on the simulator. Input data are processed by different modules, in blue, and then sent to a computer, in green, for data synchronization, visualization, and export to files. Within the system, data flows in analog (gray) or digital (black) form.

**Figure 5:**
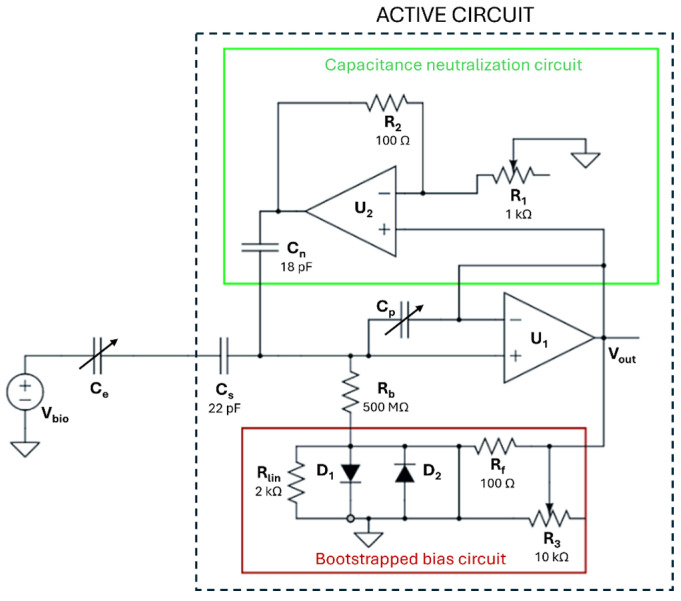
Active circuit for capacitive ECG.

The first processing module of Figure 4 is the active circuit illustrated in the schematic of Figure 5.

To measure raw biopotential V_bio_ through a capacitive skin–garment–electrode interface C_e_, a basic analog front-end design is first implemented, with the operational amplifier U_1_ in buffer configuration and, in parallel with C_e_, a bias resistor R_b_ providing the bias current required by U_1_ [52,53]. The circuit’s transfer function shows high-pass behavior, where the cut-off frequency (f_c_) depends on R_b_ and C_e_.

The basic circuit has an unavoidable parasitic capacitance C_p_ that influences f_c_ and therefore the bandwidth and frequency response of the whole analog front-end. C_p_ is neutralized by the capacitance neutralization circuit in the green box [54,55], which uses a negative impedance converter to generate a negative capacitance canceling out the parasitic input capacitance.

The capacitance C_e_ of the skin–garment–electrode interface is intrinsically variable. Changes in C_e_ can cause substantial f_c_ difference between the two textile electrodes, leading to mismatched common-mode signals and challenges to reduce noise via common-mode rejection. To reduce variability in C_e_, the only fabric allowed for the subjects’ garments in the study was cotton. The capacitance of cotton fabric was ~80 pF, as measured at 100 Hz with an E4990A Impedance Analyzer (Keysight Technologies Inc., Santa Rosa, CA, USA), following a previously described method [56]. However, the best way to reduce f_c_ variations between the two textile electrodes is to add capacitance C_s_ smaller and in series with C_e_ [42,57].

Finally, a bootstrapped bias circuit (red box) is used to provide the bias current to U_1_ while addressing the inaccuracies and leakage issues associated with ultra-high resistors like R_b_ [55,58,59]. The bias circuit provides high resistance during normal operation and a low-resistance discharge path during voltage artifacts.

The second processing module in Figure 4 is the ECG acquisition sensor SEN0213 (DFRobot/Zhiwei Robotics Corp., Shanghai, China). This Arduino-based board uses an AD8232 chip (Analog Devices Inc., Wilmington, MA, USA) as an integrated front-end that combines two signals from the active circuits into one cECG signal. Among many features, the chip notably implements:

- An instrumentation amplifier with a common-mode rejection of 80 dB and a gain of 100.
- A high-pass filter with a cutoff frequency of 1.3 Hz (blocking the DC component of the cECG) and a fast restore function reducing settling time.
- A low-pass filter with a cutoff frequency of 41 Hz and an additional gain of 5, for a total gain of 500.
- An integrated right leg drive (RLD) amplifier reinjecting the inverted common mode signal into the subject’s body via the RLD bracelet (a standard anti-static wrist strap).

The third processing module in Figure 4 is the data processing chip STM32H747ZI (STMicroelectronics NV, Plan-les-Ouates, Switzerland). This module performs a 16-bit analog-to-digital conversion of the cECG signal with a sampling rate of 500 Hz, buffers the data into the internal RAM, and transfers it to the computer via USB. An external hardware timer, working independently from the STM32H747ZI chip, was used for more accurate sampling.

The last processing module in Figure 4 is the Biopac MP160 ECG acquisition system. It takes the raw biosignals from the two Ag-AgCl reference electrodes, performs the required processing for reference ECG (rECG) acquisition, and sends the data to the computer via Ethernet. It also has its own integrated RLD amplifier reinjecting the inverted common mode signal into the subject’s body via the RLD bracelet.

No dedicated processing module is required for the Webcam PC camera. It captures images at a resolution of 640 × 480 pixels and a frame rate of 30 frames per second and sends the data to the computer via USB.

**Figure 6:**
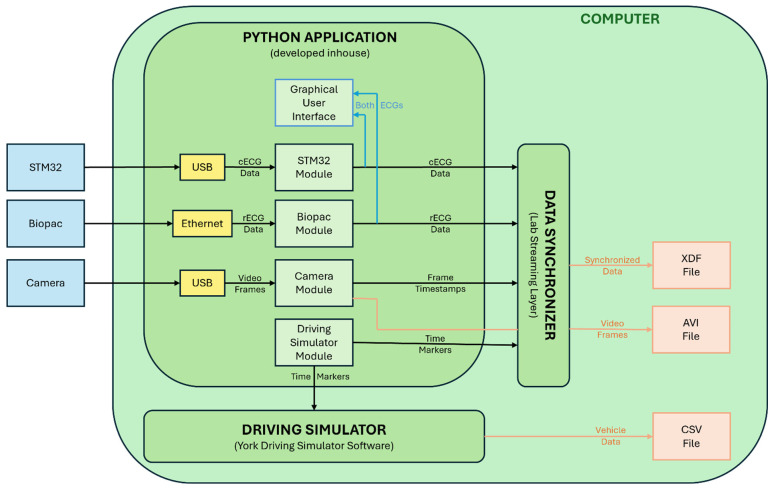
Software architecture.

The software architecture presented in the diagram of Figure 6 contains three separate applications interacting with each other in real time: a python (Python version 3.10.14) application developed in-house, a data synchronization software (Lab Streaming Layer software 1.16.2), and a driving simulator software (presented in the next section). The Lab Streaming Layer synchronization software ensures millisecond-precision temporal synchronization of data streams (see Appendix A).

The python application receives cECG data, rECG data, and video frames as inputs and performs the following tasks in parallel:

- Display cECG and rECG time series in a graphical user interface—for real-time visual inspection.
- Send a time marker to the driving simulator software and the data synchronizer every two seconds—to serve as reference for time interpolation in the data synchronization software.
- Send cECG data, rECG data, video frame timestamps, and driving simulator time markers to the data synchronization software—to put all the collected information of the study in the same timeframe.

The synchronized data are saved in an XDF file, the videos in an AVF file, and the vehicle data in a CSV file.

### 2.2. Experimental Setup

#### 2.2.1. Driving Simulator

**Figure 7:**
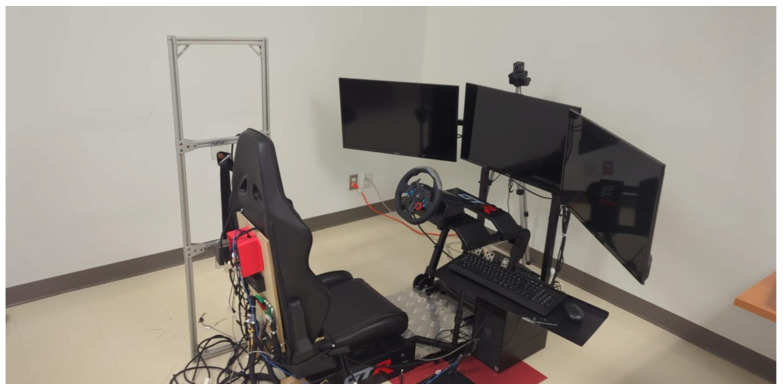
Driving simulator.

As previously described [60] and illustrated in Figure 7, an automobile’s operational environment was represented by a driving simulator with the following devices:

- Three 32-inch Samsung 1080p screens by Samsung Group (Suwon, Republic of Korea)—model UN32N5300AFXZC.
- A racing car seat by GTR Simulator (Ontario, CA, USA)—model S105L-BKRD.
- A set consisting of a steering wheel and pedals by Logitech International S.A. (San Jose, CA, USA)—model B016JBE8LU.
- A York driving simulator software by York Computer Technologies Inc. (Kingston, ON, Canada)—version 7.08.24.

#### 2.2.2. Integration of the Textile Electrodes onto the Driving Seat

**Figure 8:**
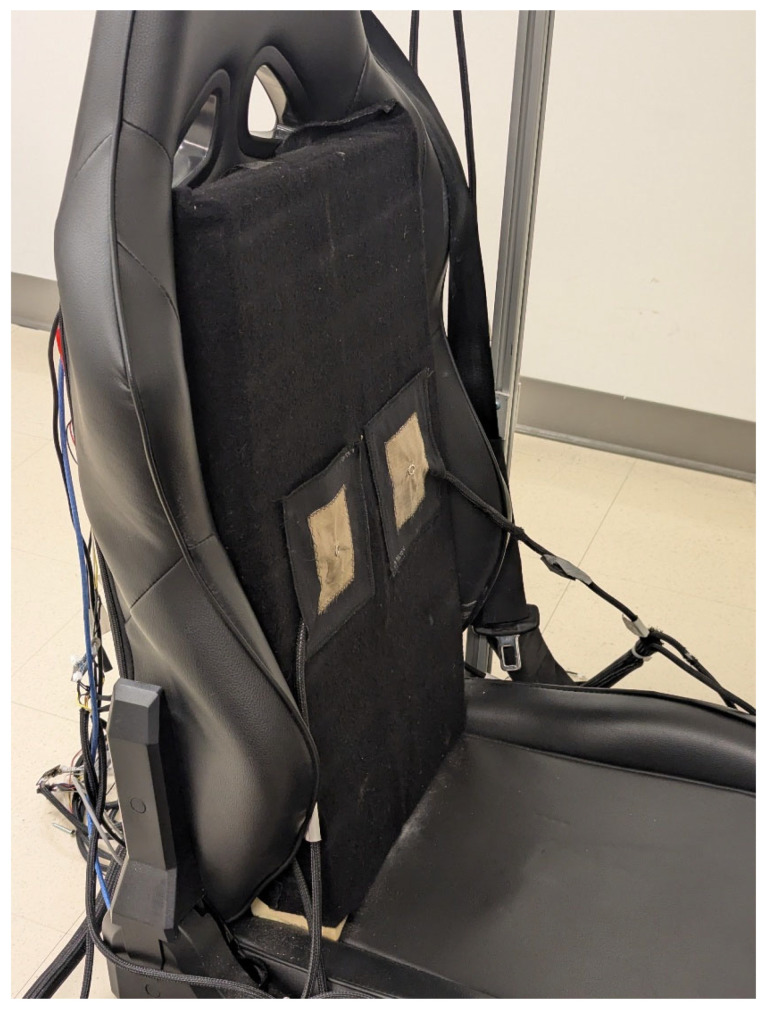
Picture of cECG textile electrodes fastened on the seat back.

As previously described [61], a VELCRO^®^ hook and loop mechanism was used to attach the electrodes to the seat at any desired position (Figure 8). Briefly, a firm polyurethane foam covered with loop fabric was placed against the back of the seat, like a cushion. Hook fabric was sewn onto the contour of the textile substrate containing the electrodes and allowed them to be fastened on the foam.

**Figure 9:**
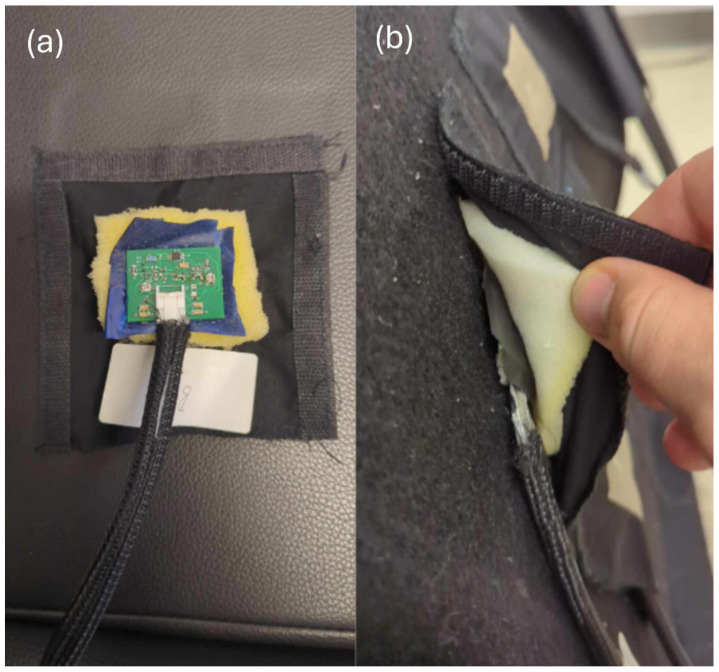
Integration of the textile electrode into the back seat. (**a**) An active circuit snapped into the back of the textile electrode. The soft foam fabric in beige and hook VELCRO^®^ at the contour of the electrode are also visible. (**b**) The electrode is partially lifted from the loop fabric covering the firm polyurethane foam to expose the active circuit, the soft foam, and the hook VELCRO^®^.

The signal acquisition circuit was connected to the back of the textile electrode with the stud part of a snap-in connector, and a soft foam padding was included for improved support (Figure 9).

**Figure 10:**
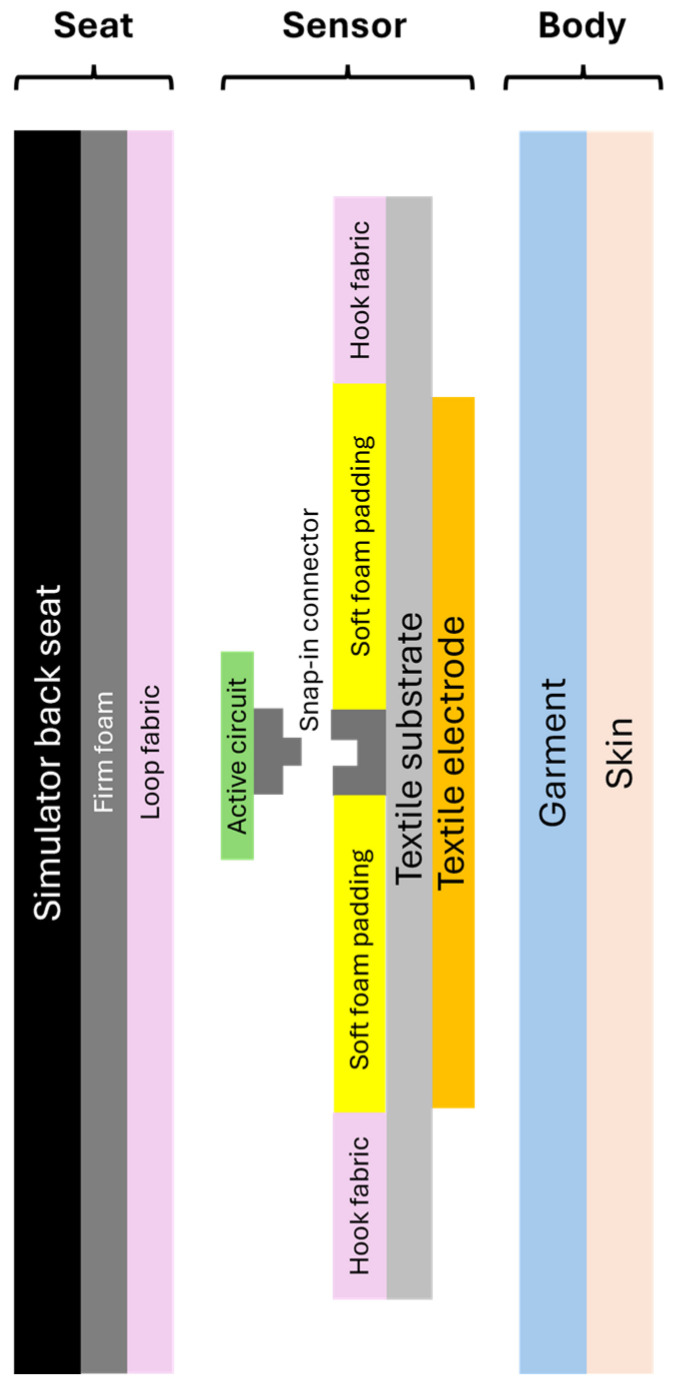
Schematic of all the layers between the simulator seat back and the skin.

Figure 10 shows all the layers of material between the driving simulator seat and the skin. From left to right, there are 3 blocks: the seat, the sensor, the body. The seat contains the simulator back seat with the firm foam covered with loop fabric. The sensor first consists of the textile electrode embroidered on a textile substrate with hook fabric on its contour, soft foam padding and the socket part of snap-in connector. The other component of the sensor is the active circuit with the stud part of the snap-in connector. Finally, the subject’s body is represented in the schematic by the garment and the skin.

### 2.3. Data Acquisition

#### 2.3.1. Selection of Subjects

Data were collected on 7 healthy subjects in their twenties. All the subjects were male, except subject #4 who was female. Three inclusion criteria were considered: the ability to drive, good general health, and no known heart pathology. To characterize subject’s morphology several measurements were taken, notably the height, the torso length (distance between the top of the sternum and the belly button), and three circumferences: bustline, waistline, shoulder line (see Table 1). Subjects’ morphologies included in the study were varied to account for a large diversity of body sizes.

#### 2.3.2. Acquisition Protocol

**Figure 11:**
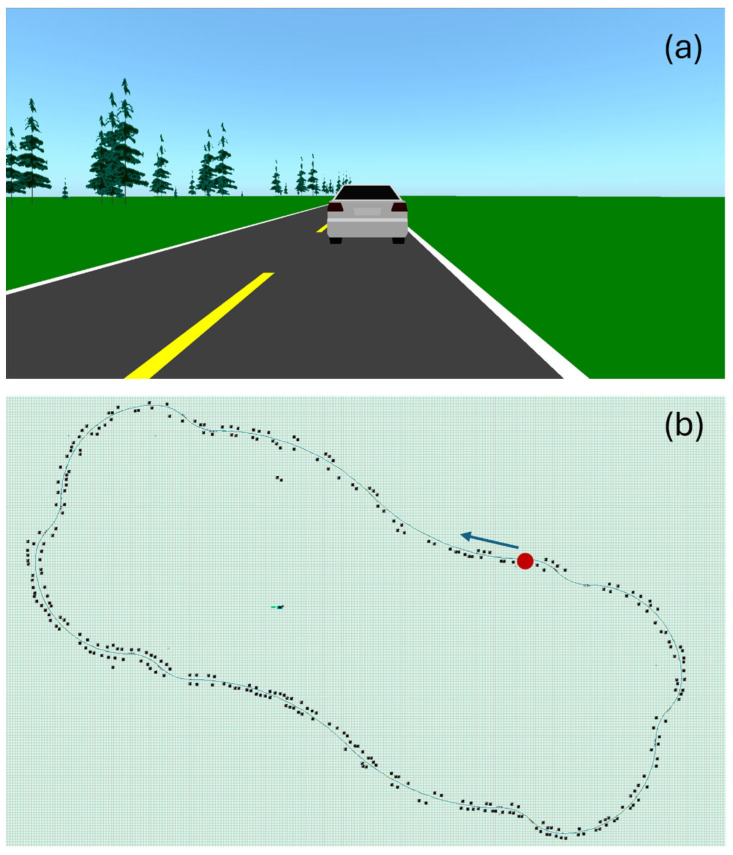
Print screens of the driving simulator’s user interface. (**a**) Scenery. The road is bordered with greenery, including trees. While driving, the subject is following a car. (**b**) Driving circuit. The green line represents the road. The black dots are trees. The circuit starts at the red dot, in the direction of the blue arrow. Picture and caption reproduced from [60], distributed under the terms and conditions of the Creative Commons Attribution (CC BY) license.

For each data acquisition session, the subject followed the driving protocol, shown in Figure 11. The subject follows a vehicle leading at 100 km/h, alternating mostly slow curves left and right. The driving circuit was divided into 20 segments with different radiuses of curvature (Table 2), for a total length of 14.6 km. There are no straight segments —the driver is consistently making micro-adjustments. It takes approximately 9.5 min to complete the circuit. A 15 min break was granted each time two circuits were completed, to prevent attention fatigue. This protocol simulates the maximum legal highway speed in Quebec, Canada, while incorporating established driving procedures for drowsiness detection. Indeed, monotonous driving simulations are commonly used in drowsiness detection research, especially as monotony, characterized by quite straight roads, low traffic, and minimal stimulation, leads to cognitive underload, reduced alertness, and fatigue. This type of environment on a simulator effectively replicates real-world high-risk scenarios such as long-distance trips and highways, allowing controlled and repeatable assessments of drowsiness [62,63,64,65].

**Figure 12:**
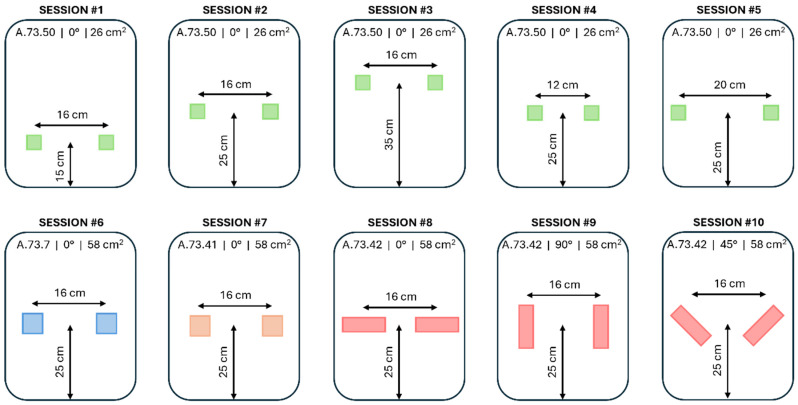
Sessions of acquisition. The subject performed the complete circuit 10 times, once for each acquisition session.

Each subject was asked to perform the complete driving circuit 10 times, corresponding to the 10 acquisition sessions of Figure 12 and Table 3. Each session corresponded to a pair of textile electrodes with a different combination of placement and design. According to Figure 12, the height is the distance between the seat of the car seat and the center of an electrode. The spacing is the distance between the center of the electrodes. The shape of the electrodes can be square (S) or rectangle (R). The angle can be 0°, 45°, or 90°, with 0° corresponding to the horizontal rectangle; all square-shaped electrodes were considered at 0°. Surface areas of 26 cm^2^ and 58 cm^2^, with woven (W) or embroidered (E) fabric were also considered in this study.

In these experiments, we systematically vary the height and spacing of the electrodes to increase spatial coverage across a larger and more diverse population, addressing the limitations of previous studies that focused on lumbar or thoracic placements with smaller sample sizes [21,33,39,40,43,49,50]. Building on research on the trade-off between textile cECG electrode size and signal quality [24,41], we use comparable electrode dimensions while incorporating embroidered electrodes and slight size adjustments to investigate accommodation to different body morphologies. Rectangular electrodes are tested at 90° to assess proximity to the heart and at 45° to simulate angular configurations approximating a hexaxial reference system.

For each session, the subject was seated with the seat belt on, and was instructed to focus on driving, with no other movement allowed. Signals containing cECG and rECG data, and video images were taken during each session, simultaneously and independently. The whole study generated 70 textile cECG signals, 70 reference rECG signals, and 70 video recordings.

### 2.4. Data Analysis

The calculations in this study were performed with MatlabR2024b^®^. All rECG and cECG signals were downsampled at 125 Hz and standardized using a z-score normalization. After a visual inspection of the whole cECG data set, all amplitudes above +8 or below −8 were considered high amplitude spikes due to amplifiers’ saturation and capped at ±8.

#### 2.4.1. Measurement of cECG RR Durations

**Figure 13:**
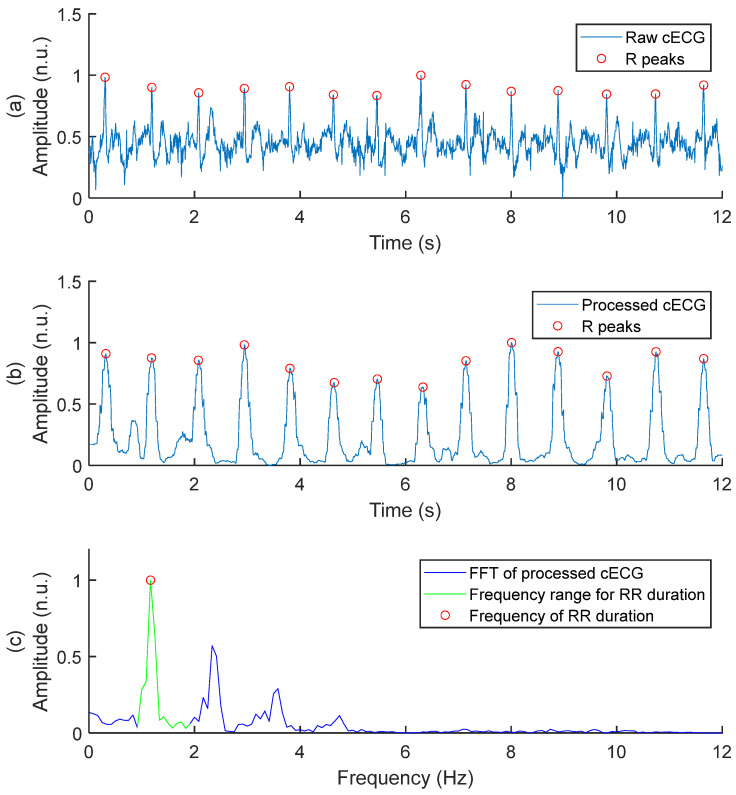
FFT-based method to measure cECG RR duration. (**a**) Raw cECG signal. To improve visualization, baseline wander has been removed by subtracting a moving average with a window of 125 samples and a step of 31 samples. (**b**) Processed cECG inspired by the Pan–Tompkins algorithm. (**c**) FFT of processed cECG, with the frequency range for RR duration and the frequency corresponding to RR duration.

An FFT-based (Fast Fourier Transform) method has been developed in-house to measure RR durations. The method is illustrated in Figure 13. The raw cECG in panel (a) is processed into the signal of panel (b), using a method inspired from the Pan–Tompkins (PT) real-time ECG QRS detection algorithm [66]:

1. A [5–15 Hz] band-pass filter is applied to enhance the QRS complex and attenuate any other feature of the cECG. To do so, the FFT of the cECG signal is performed. The amplitude of all frequencies outside of the range [5–15 Hz] is put to zero. Then, the inverse FFT of the spectrum is performed to obtain the filtered cECG signal in the time domain.
2. The amplitude of the filtered cECG is clipped at the 95th percentile to eliminate the highest amplitudes, but without eliminating the QRS complexes.
3. The first derivative of the filtered cECG signal is approximated simply by calculating the difference between cECG points at time *t* and time *t − 1*.
4. Squaring is applied to all data points of the signal from step 3.
5. A moving average filter is applied to the signal from step 4. The filter is implemented with a window of 19 points moving with a step of 10 points. The averaged point is at the center of the window.

The FFT of the processed cECG is performed, as illustrated in Figure 13c. For the study, the range of interest for cardiac frequency is [0.833–1.833 Hz], corresponding to a resting heart rate of [50–110 bpm] in a healthy subject. The frequency corresponding to the cECG RR duration is the frequency with the highest amplitude within the range of interest.

For verification purposes, the rECG RR durations were measured with the FFT-based method, and compared with rECG RR durations measured with a MatlabR2024b^®^ implementation of the PT-based algorithm [67]. The success criterium was for the FFT-based and PT-based algorithms to measure the same rECG RR durations. First, the PT-based algorithm was used to detect the R peaks from the 70 rECG signals of the study. The peaks were visually inspected to ensure 100% accuracy. Peak-to-peak RR durations were obtained by calculating the successive differences between R peak time positions. The PT-based RR duration for a whole signal was calculated as the average of peak-to-peak RR durations for that signal. Second, the FFT-based method, applied to the whole rECG signal, was used to measure the RR durations for all 70 rECG signals of the study. Finally, the RR durations obtained with PT-based and FFT-based algorithms were compared by performing a linear regression, and computing Pearson correlation and average error.

For further verification, errors on cECG RR durations were evaluated for the FFT-based versus the PT-based method. The benchmark to calculate the error was the PT-based RR durations on rECGs. The success criterium was for the FFT-based method to have lower average errors compared to the PT-based method.

#### 2.4.2. Assessment of cECG Signal Quality

**Figure 14:**
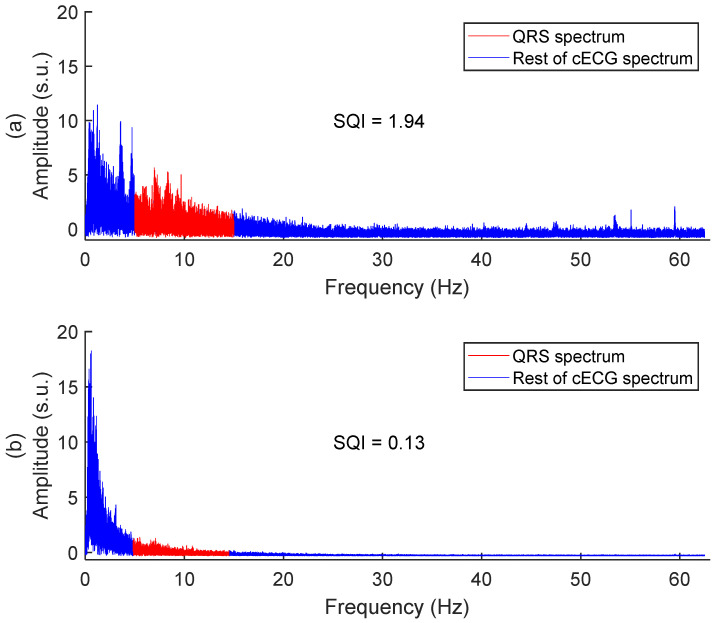
Examples of textile signal SQI with corresponding spectrums. (**a**) Good quality signal, taken on subject #2 in session #5. (**b**) Poor quality signal, taken on subject #1 in session #6. Amplitudes are in standard score units.

We introduced a signal quality index (SQI) based on the relative power of the QRS complex. The SQI calculates the ratio of the power in the QRS frequency range (5 Hz to 15 Hz) to the power of the rest of the cECG spectrum. Figure 14 shows two examples of the SQI with corresponding spectrums.

Every cECG and rECG signals of the study were segmented into 35 sections of 15 s, with no overlap. The SQI calculation was applied to each 15-s section.

To make a general assessment of signal quality throughout the study, cECGs were compared to rECGs. For each cECG or rECG signal, the 35 SQI values were averaged, leading to one SQI value per signal. The cECG and rECG SQIs were pooled in two groups, and the null hypothesis was tested with the binomial sign test, with the significant *p* value set at 0.05.

To compare the signal quality of a cECG group A versus a cECG group B, we first dispatched the appropriate SQI values between groups A and B. The null hypothesis was tested with the binomial sign test, with the significant *p* value set at 0.05. Table 4 summarizes the objectives of each experimental test conducted to investigate how variations in electrode placement and design influence signal quality.

#### 2.4.3. Assessment of the Accuracy of RR Duration Measurements

Accuracy is assessed by comparing cECG RR durations to rECG RR durations. Similarly to the assessment of signal quality, every cECG and rECG signal in the study were segmented into 35 sections of 15 s, with no overlap. The FFT-based RR duration measurement was applied to each 15-s section. Then, error (ERR) and error in percentage (ERP) were calculated as follows:

(1) $$ERR={RR}_{reference}-{RR}_{textile}$$

(2) $$ERP=\frac{{RR}_{reference}-{RR}_{textile}}{{RR}_{reference}}$$

The 35 ERR values were averaged for each signal, leading to one ERR value per cECG. The process is repeated for ERP. To make a general assessment of accuracy throughout the study, three approaches were taken. First, the ERP distribution was calculated to describe how the RR duration errors are spread across the whole study. Second, a Bland–Altman plot was generated to quantify the agreement between each RR duration measured on a cECG signal and the corresponding RR duration measured on a rECG signal. Finally, the error coverage rate was calculated as the percentage of data within a certain range of ERP. More specifically, for each cECG signal, the error coverage rate within [0, 5] is the percentage of the 35 calculated ERP values falling between 0% and 5%. For the whole study, the average of the 70 error coverage rates within [0, 5] was taken. The process was repeated to calculate error coverage rates for the ranges: ]5, 10], ]10, 15], ]15, 20], ]20, ∞[.

## 3. Results

### 3.1. General Assessment of Signal Quality

**Figure 15:**
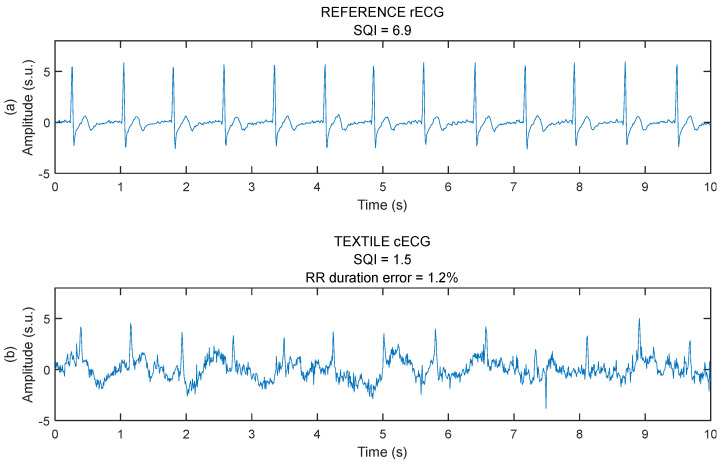
Samples of raw ECG signals. (**a**) Reference rECG taken with Ag-AgCl electrodes glued on the chest’s skin. (**b**) Textile cECG taken through a one-layer cotton garment with textile capacitive electrodes attached to the car seat. Both signals were simultaneously acquired from subject #6 in session #5.

Samples of the rECG and cECG are shown in Figure 15. rECG has a better signal quality compared to the cECG, with a higher SQI. The higher SQI is explained by the absence of baseline wander, well-defined QRS complexes, and low levels of high frequency noise. However, the RR duration error on cECG is still low at 1.2%. This result, where cECG displays a higher level of noise and still a low RR duration error, is consistent throughout the study.

**Figure 16:**
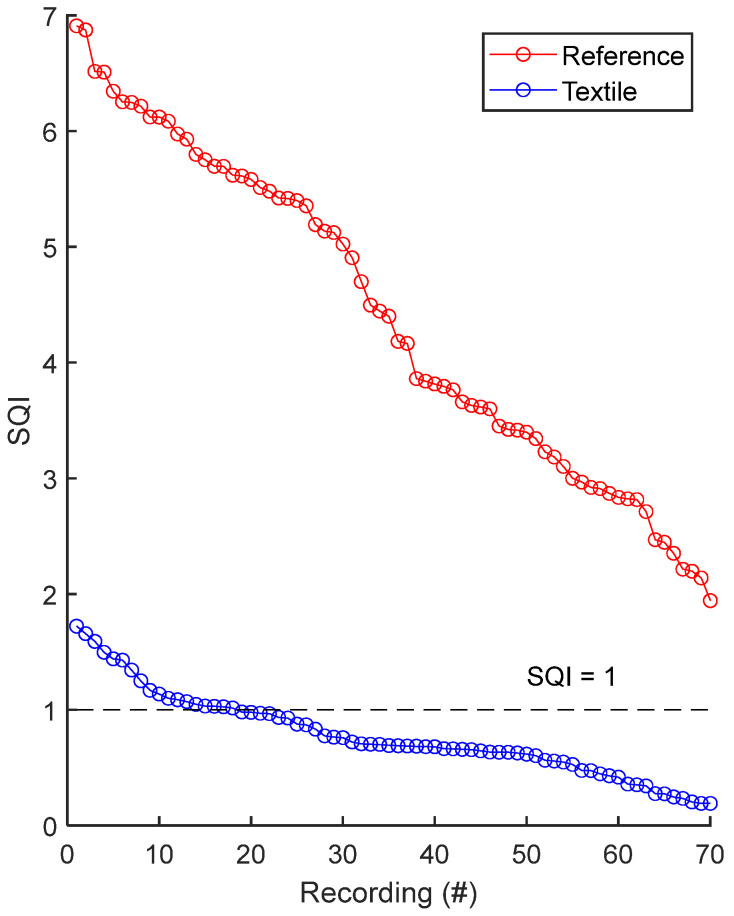
Signal quality index: reference rECG vs. textile cECG. SQIs are sorted in decreasing order and plotted for reference and textile electrodes. There is one dot for each rECG or cECG signal recording. The value of one dot is the average of 35 SQIs values calculated on 35 fifteen-second sections of signal. The dashed line at SQI = 1 shows the threshold below which the QRS complex has a lower power compared to the rest of the ECG spectrum.

As shown in Figure 16 and Table 5, rECGs displayed higher quality compared to cECGs. The SQI of the rECG is consistently higher compared with cECG, and also consistently higher than one. The SQI difference between the two groups is statistically significant. Furthermore, 74% of cECG signals had an SQI lower than one, meaning more observable noise compared to rECG.

### 3.2. Verification of the FFT-Based Method for Measuring RR Duration

**Figure 17:**
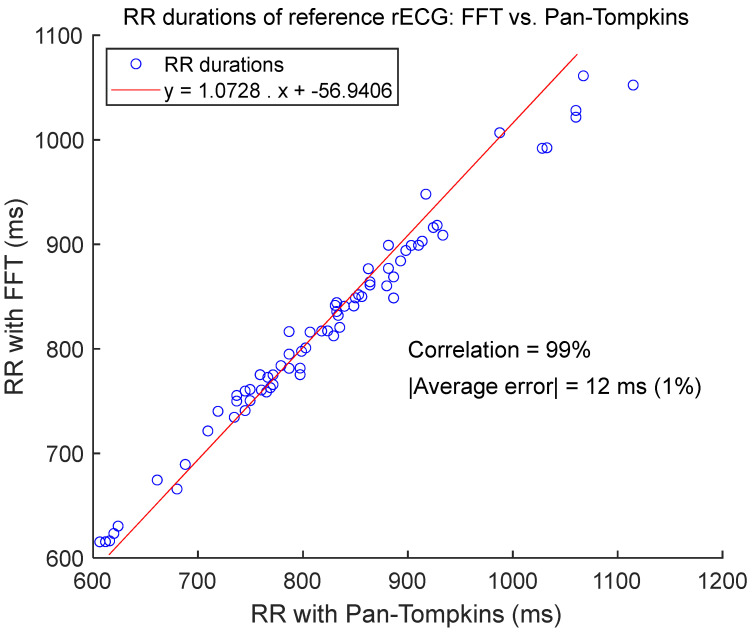
RR durations of rECG signals: FFT-based method vs. PT-based method. There is one dot for each rECG signal −70 dots in total. The value of each dot is the RR duration calculated with the FFT-based method versus the PT-based method. Each method is applied to the whole rECG signal. The red line is the linear regression of the data set.

As illustrated in Figure 17, the FFT-based method for measuring RR duration showed similar performance compared to the gold standard PT-based method, when applied to rECGs. The two sets of RR durations were linearly dependent, with a slope of 1.07 and a correlation coefficient of 99%. The average error across the 70 rECG signals was 12 ms or 1%.

**Figure 18:**
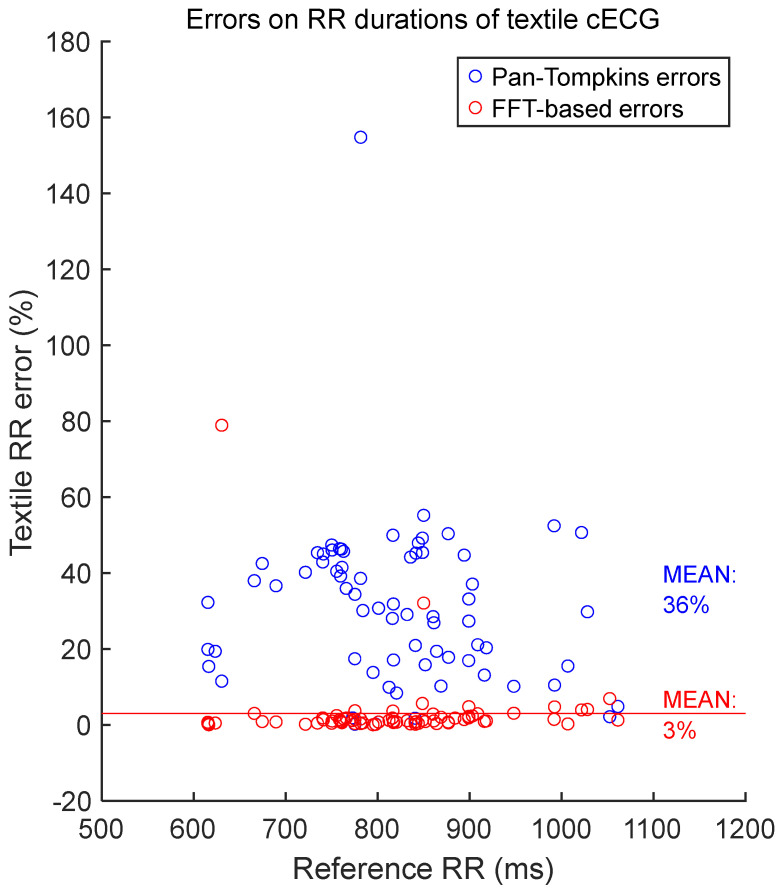
Errors on RR durations measured on textile cECG: FFT-based method vs. PT-based method.

RR durations measured on cECG with the FFT-based method were compared with the gold standard RR durations, measured on rECGs with the PT-based method. The average error was 3%, as shown in Figure 18. However, RR durations measured on cECG with the PT-based algorithm displayed an average error of 36%.

### 3.3. General Assessment of the Accuracy of RR Durations Measurements

**Figure 19:**
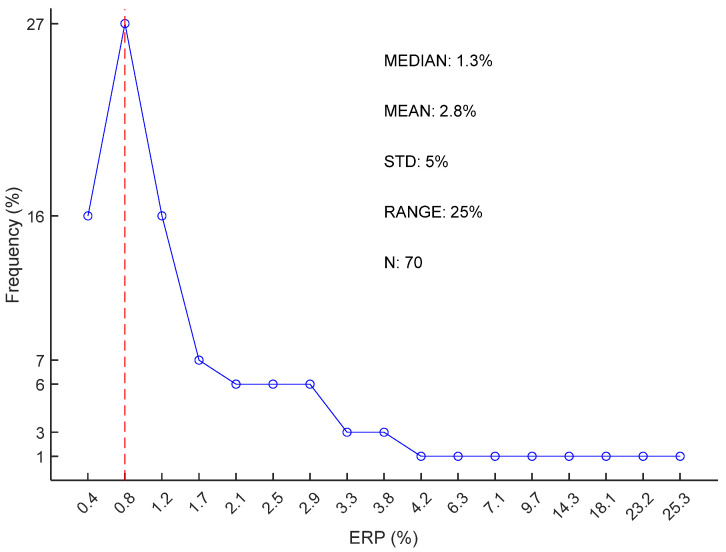
cECG RR duration accuracy with the FFT-based method: distribution ERP.

According to Figure 19, 27% of cECG RR durations were accurate at approximately ERP = 0.8%. The mean ERP for the whole study was 2.8% with a standard deviation of 5%. However, the range of 25% indicated the presence of outliers.

**Figure 20:**
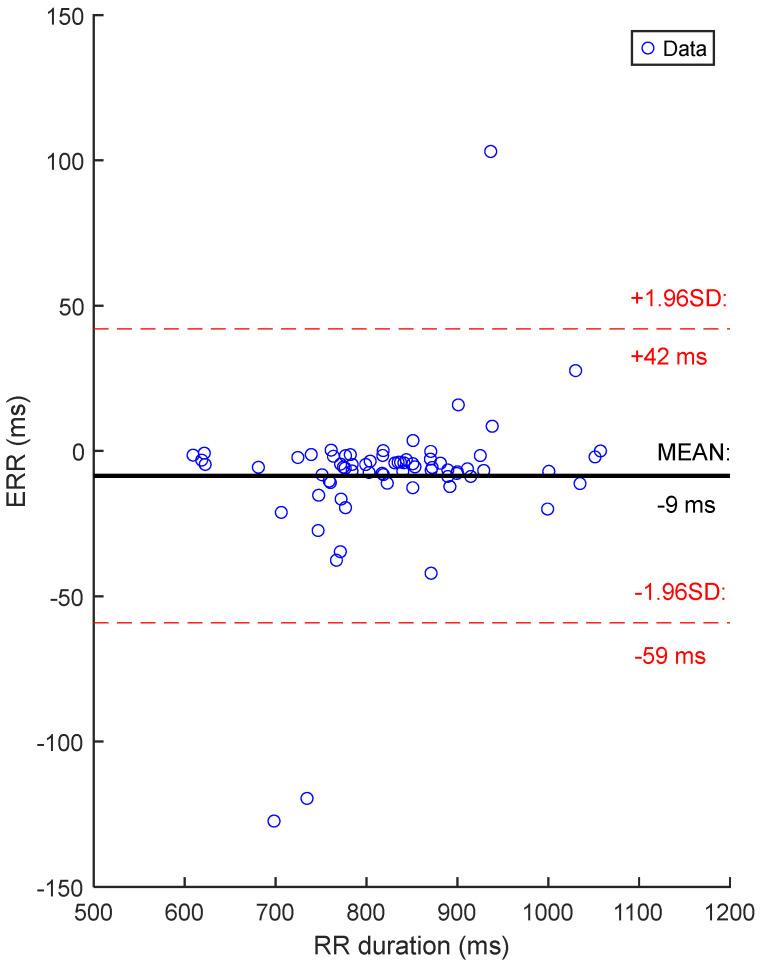
Bland–Altman plot of RR duration errors with the FFT-based method: cECG vs. rECG. There is one dot for each textile cECG signal, for a total of 70 signals. The value of one dot is the average of 35 ERR values calculated on 35 15-s sections of signal.

The Bland–Altman plot of Figure 20 demonstrated that the systematic bias, expressed with the mean error between cECG and rECG RR durations, is −9 ms. Most measurements stayed close to the average error, within the limits of agreement of 1.96 times the standard deviation, with 3 outliers over 70 data points.

According to Table 6, on average, each cECG signal maintained an ERP between 0% and 5% during 81% of the recording. The coverage rate quickly collapsed for higher ERPs.

### 3.4. Relationship Between Signal Quality and Accuracy

**Figure 21:**
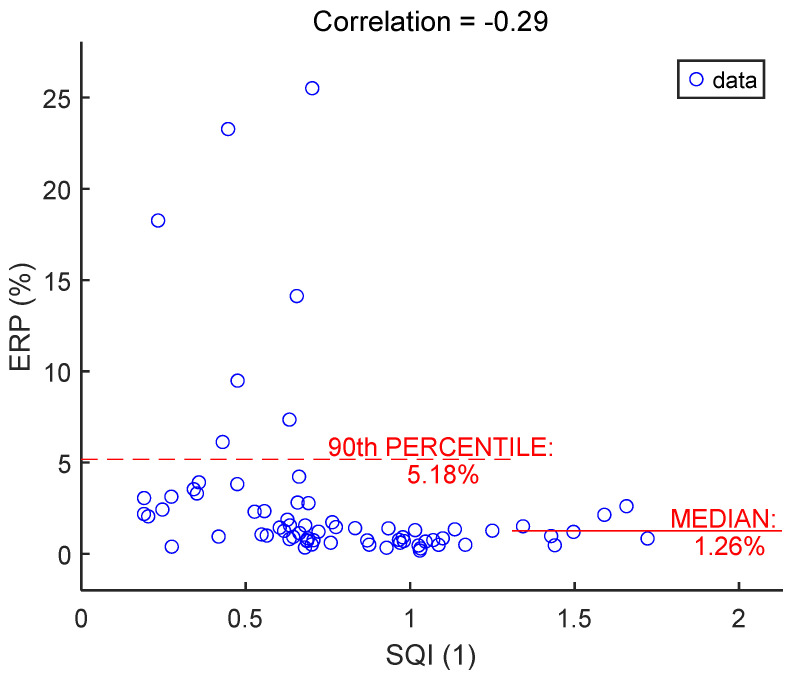
RR duration percentage error vs. signal quality index. There is one dot for each textile cECG signal, for a total of 70 signals. The value of one dot is the average of 35 ERP values calculated on 35 15-s sections of signal.

As illustrated in Figure 21, large errors and outliers occurred at low SQIs, typically below 0.7. However, 90% of errors were below 5%, independent of SQI.

### 3.5. Effect of Electrode Position on Signal Quality

**Figure 22:**
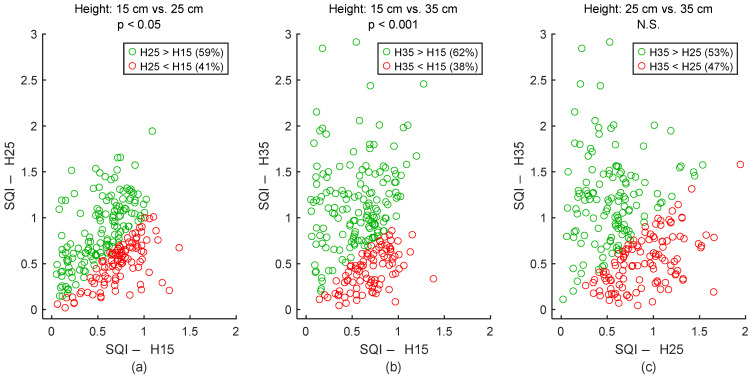
Effect of electrodes’ height on signal quality. (**a**) 15 cm vs. 25 cm. (**b**) 15 cm vs. 35 cm. (**c**) 25 cm vs. 35 cm. Each dot represents a pair of SQI values from the same person but two different electrodes’ heights. The two SQI values are calculated on a 15-s section of two different cECG signals but within the same timeframe of driving simulation. There are 245 dots corresponding to the 245 sections of a cECG signal: 7 subjects × 1 signal per subject × 35 sections per signal. H15, H25, and H35 are abbreviations for “Height: 15 cm”, “Height: 25 cm”, and “Height: 35 cm”, respectively.

To assess signal quality as a function of electrodes’ height, textile cECGs from sessions #1, #2, and #3 (Table 4: Comparison I) were analyzed for the seven subjects. SQI values were dispatched into three groups: 15 cm, 25 cm, and 35 cm. Results are shown in Figure 22 and Table 7. Signal quality was directly proportional to electrodes’ height on the back. In fact, according to the average SQI, 15 cm was outperformed by both 25 cm and 35 cm. Differences were statistically significant. In average SQI, 25 cm was also outperformed by 35 cm, but the difference was not significant.

**Figure 23:**
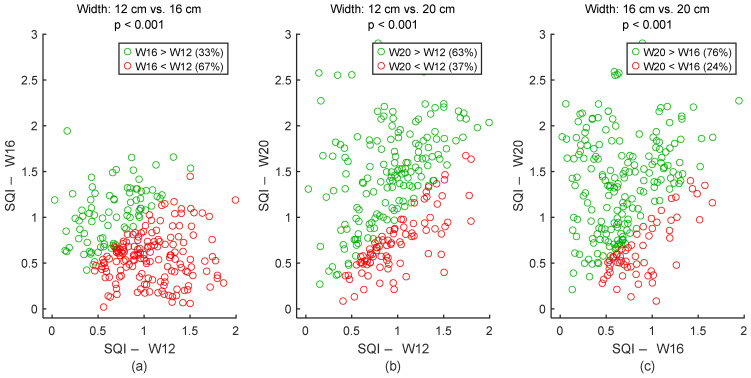
Effect of spacing between electrodes on signal quality. (**a**) 12 cm vs. 16 cm. (**b**) 12 cm vs. 20 cm. (**c**) 16 cm vs. 20 cm. Each dot represents a pair of SQI values from the same person but two different electrodes’ widths. The two SQI values are calculated on a 15-s section of two different cECG signals but within the same timeframe of driving simulation. There are 245 dots corresponding to the 245 sections of a cECG signal: 7 subjects × 1 signal per subject × 35 sections per signal. W12, W16, and W20 are abbreviations for “Width: 12 cm”, “Width: 16 cm”, and “Width: 20 cm”, respectively.

To assess signal quality as a function of electrodes’ spacing, textile cECGs from sessions #4, #2, and #5 (Table 4: Comparison II) were analyzed for the seven subjects. SQI values were dispatched into three groups: 12 cm, 16 cm, and 20 cm. Results are shown in Figure 23 and Table 8. According to the average SQI, 20 cm outperformed both 16 cm and 12 cm. However, 12 cm displayed better outcome compared to 16 cm. All differences were statistically significant.

**Figure 24:**
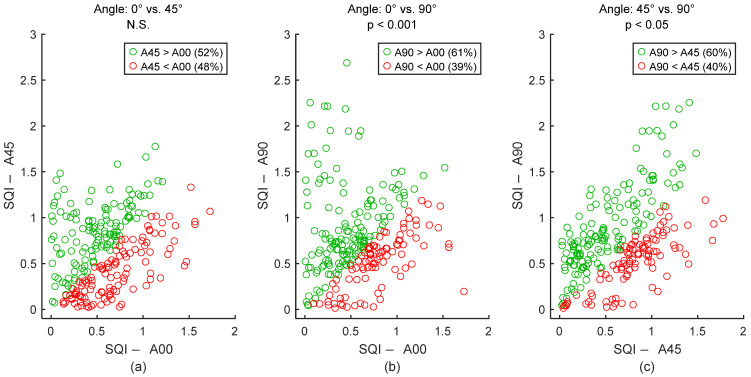
Effect of electrodes’ angle on signal quality. (**a**) 0° vs. 45°. (**b**) 0° vs. 90°. (**c**) 45° vs. 90°. Each dot represents a pair of SQI values from the same person but two different electrodes’ angles. The two SQI values are calculated on a 15-s section of two different cECG signals but within the same timeframe of the driving simulation. There are 245 dots corresponding to the 245 sections of a cECG signal: 7 subjects × 1 signal per subject × 35 sections per signal. A00, A45, and A90 are abbreviations for “Angle: 0°”, “Angle: 45°”, and “Angle: 90°”, respectively.

To assess signal quality as a function of the electrodes’ angle, textile cECGs from sessions #8, #10, and #9 (Table 4: Comparison III) were analyzed for the seven subjects. SQI values were dispatched into three groups: 0°, 45°, and 90°. Results are shown in Figure 24 and Table 9. Signal quality was directly proportional to the electrode’s angle on the back. In fact, according to the average SQI, 90° outperformed both 45° and 0°, with statistically significant differences. The angle 45° displayed a better outcome compared to 0°, but the difference was not significant.

### 3.6. Effect of Electrode Form Factor on Signal Quality

**Figure 25:**
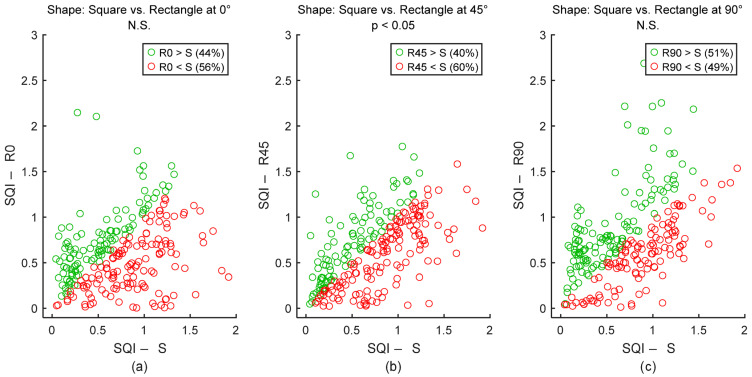
Effect of electrodes’ shape on signal quality. (**a**) Square vs. Rectangle at 0°. (**b**) Square vs. Rectangle at 45°. (**c**) Square vs. Rectangle at 90°. Each dot represents a pair of SQI values from the same person but two different electrodes’ shapes. The two SQI values are calculated on a 15-s section of two different cECG signals but within the same timeframe of driving simulation. There are 245 dots corresponding to the 245 sections of a cECG signal: 7 subjects × 1 signal per subject × 35 sections per signal. S, R0, R45, and R90 are abbreviations for “Square”, “Rectangle at 0°”, “Rectangle at 45°”, and “Rectangle at 90°”, respectively.

To assess signal quality as a function of electrodes’ shape, textile cECGs from sessions #7, #8, #10, and #9 (Table 4: Comparison IV) were analyzed for the seven subjects. SQI values were dispatched into four groups: Square and Rectangle at 0°, 45°, and 90°. Results are shown in Figure 25 and Table 10. Square electrodes had better signal quality compared to Rectangle at 0° and 45°. In fact, according to the average SQI, Square outperformed both Rectangle at 0° and Rectangle at 45°, with a statistically significant difference with Rectangle at 45°. In average SQI, Square was outperformed by Rectangle at 90°, but the difference was not significant.

**Figure 26:**
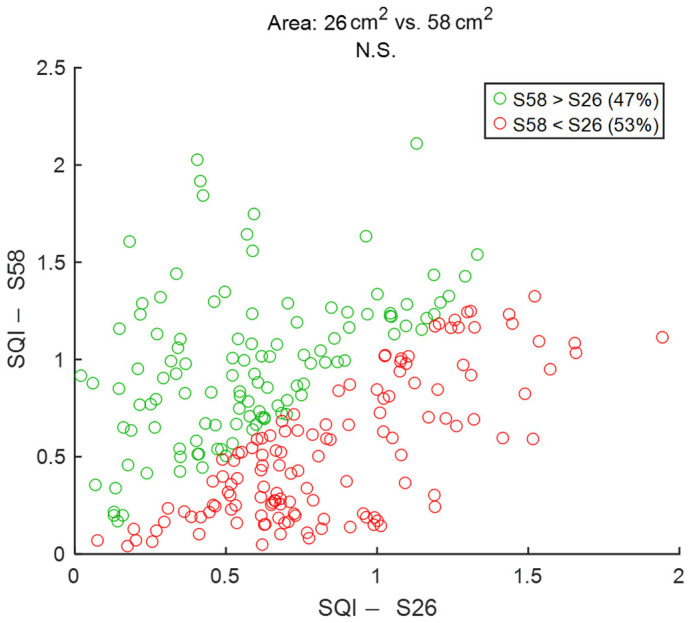
Effect of electrodes’ size on signal quality: 26 cm^2^ vs. 58 cm^2^. Each dot represents a pair of SQI values from the same person but two different electrodes’ surface areas. The two SQI values are calculated on a 15-s section of two different cECG signals but within the same timeframe of driving simulation. There are 245 dots corresponding to the 245 sections of a cECG signal: 7 subjects × 1 signal per subject × 35 sections per signal. S26 and S58 are abbreviations for “Area: 26 cm^2^” and “Area: 58 cm^2^”, respectively.

To assess signal quality as a function of electrodes’ size, textile cECGs from sessions #2 and #7 (Table 4: Comparison V) were analyzed for the seven subjects. SQI values were dispatched into two groups: 26 cm^2^ and 58 cm^2^. Results are shown in Figure 26 and Table 11. Both groups displayed similar performances with almost equal average SQIs and a non-significant difference.

### 3.7. Effect of Electrode’s Fabric Type on Signal Quality

**Figure 27:**
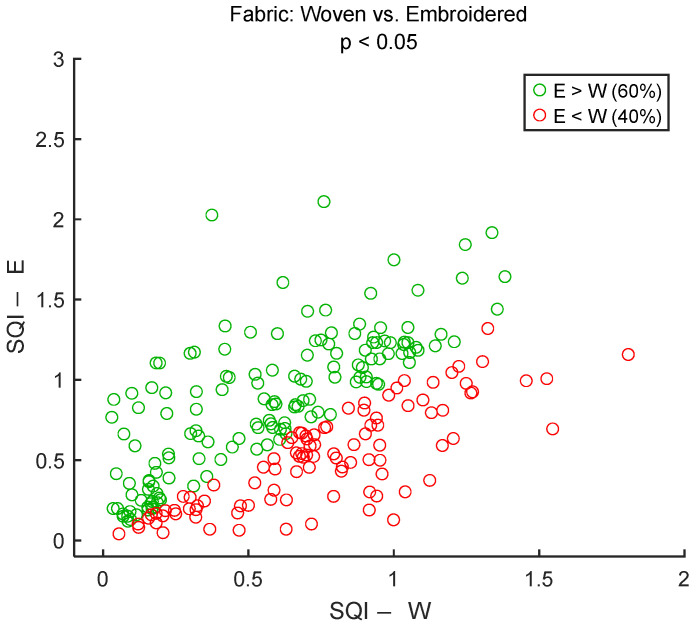
Effect of electrodes’ fabric on signal quality: Woven vs. Embroidered. Each dot represents a pair of SQI values from the same person but two different electrode’s types of fabric. The two SQI values are calculated on a 15-s section of two different cECG signals but within the same timeframe of driving simulation. There are 245 dots corresponding to the 245 sections of a cECG signal: 7 subjects × 1 signal per subject × 35 sections per signal. W and E are abbreviations for “Fabric: Woven” and “Fabric: Embroidered”, respectively.

To assess signal quality as a function of electrodes’ fabric type, textile cECGs from sessions #6 and #7 (Table 4: Comparison VI) were analyzed for the seven subjects. SQI values were dispatched into two groups: Woven and Embroidered. Results are shown in Figure 27 and Table 12. According to the average SQI, Embroidered outperformed Woven. The difference was statistically significant.

## 4. Discussion

### 4.1. Summary and Interpretation of the Results

In this study, we presented a capacitive ECG acquisition system using textile electrodes. We assessed the effects of electrodes’ design and placement during driving simulations involving seven participants of varied morphologies.

cECG signals with observable R peaks could be captured with almost all pairs of textile electrodes, independently of design or placement, after appropriate filtering. This indicates the existence of design and placement freedom for applications requiring only R peaks, notably to calculate heart rate variability for applications such as drowsiness detection in driving conditions [4].

Signal quality in textile cECGs was lower compared to rECG from reference Ag-AgCl electrodes. Most of the time, filtering was needed to clearly observe cECG R peaks, contrary to rECGs. However, low signal quality did not prevent the accurate measurement of RR durations in cECGs compared to rECGs. The accuracy in RR duration measurement was algorithm-dependent, where the classic PT-based algorithm was interestingly outperformed by an in-house FFT-based algorithm. This indicates there is a trade-off to be made between freedom of design and placement on one hand, and freedom of the algorithm, on the other hand. For applications where only time-domain peak detection methods like Pan–Tompkins are available, it becomes important to pick designs and placements maximizing signal quality.

Proximity to the heart or even contact with the body seems to be the key to improving quality. For example, the SQI from Square electrodes on upper back (height = 35 cm) outperforming lower back illustrated the importance of proximity to the heart. The SQI from Rectangular electrodes at 90° (vertical, along the back muscles) outperforming 0° (horizontal, across the back muscles) indicated how an even contact with the body can make a significant difference.

However, not all designs provided such intuitive results and simple interpretations. For example, we observed no particular effect of electrode size on signal quality. Larger electrodes theoretically enhance signal intensity due to increased coupling capacitance. However, in the same manner as reported in previous studies [24], we also did not observe any consistent improvement in noise levels with increasing electrode area. Noise from external electrical sources, body motion, and inconsistent electrical interference likely affect the outcome. Variations in body shapes may also affect signal quality, as this parameter can influence mechanical contact with electrodes, potentially reducing the effective electrode area and impacting signal collection [24]. In addition, a comprehensive model of electronic noise and frequency responses for cECG textile electrodes [68] observed that only a significant increase in electrode surface area intensifies 1/f-power noise.

Some results need further investigations before any interpretations can be made. For example, no direct proportionality was found between electrodes’ spacing on the back and signal quality. The spacing of 20 cm and 12 cm outperforming 16 cm may indicate the impact of the subjects’ back morphology on the effective surface area of the electrode. The observed signal quality variations as a function of electrode spacing can be attributed to a combination of capacitive coupling efficiency and electromagnetic interference effects. Capacitive ECG monitoring relies on the interface between the electrodes and the heart via body tissues, which exhibit a non-linear relationship with electrode spacing [41,69,70]. A spacing of 20 cm and 12 cm appears to optimize this coupling, enhancing signal transmission and amplitude given the wide variety of subjects’ morphologies. Conversely, 16 cm spacing may fall within zones more susceptible to electromagnetic interference, where interactions between the body’s bioelectric field and external sources can degrade signal quality [71,72]. This interference is frequency-dependent and influenced by electrode positioning [73]. As highlighted by some previous studies, electrode spacing affects both signal strength and noise sensitivity [41,69,74]. The 20 cm or 12 cm configuration likely avoids critical interference zones while maintaining effective coupling, resulting in a better signal quality compared to 16 cm spacing. These observations underline the importance of empirically optimizing electrode placement in cECG systems, as even small adjustments in spacing can significantly impact signal quality due to complex electromagnetic interactions.

Our observations about embroidered cECG electrodes are consistent with those observed on galvanic textile electrodes. Due to their larger contact area, better signal stability during movement, and greater mechanical durability, embroidered ECG electrodes generally provide superior performance compared to woven electrodes for wearable heart monitoring [75,76,77]. However, more explorations, including the electromechanical and physical characterization of textile cECG electrodes, are also needed to better explain why embroidered fabric displayed higher cECG SQIs compared to woven fabric.

### 4.2. Originality and Relevance

This paper brings several research innovations, notably in the electrode fabrication process (application of industrial embroidery technology), experimental design (multi-electrode configuration and diverse subjects), and algorithm development (independently developed frequency-domain algorithm). To our knowledge, this study is the first to specifically explore the effect of textile capacitive electrodes’ design and placement during driving simulations.

The textile capacitive electrode developed in this study can be directly integrated into the backrest of automotive seats, providing a low-cost and high-comfort heart rate measurement solution for on-board health monitoring systems, which is conducive to identifying early warnings of drowsiness while driving. We involved seven participants of varied morphologies to ensure generalizable results. The FFT-based method for measuring RR duration is a good alternative to the PT-based method for all applications where the cECG signal is noisy and where enough computational power is available. The trade-off between freedom of design and freedom of algorithm featured in this paper can help inform the design process by optimizing resource management based on the product’s use cases and constraints. The superior signal quality achieved with embroidered electrodes compared to woven ones highlights their potential as better candidates for integration into vehicle seats.

### 4.3. Limitations

According to the present study, the first limitation of textile capacitive electrodes is the low signal quality. If the end goal of a cECG system is only to gather R peaks, this is not an issue. But acquiring complete cECGs with observable P and T waves would be challenging. Several studies already address noise at the circuit level [37,78,79]. Adapting those circuits to the specific needs of textile electrodes, like correcting the mismatched common-mode signals between the two electrodes and therefore increasing the possibility of reducing noise via common-mode rejection, could help improve the signal quality.

All subjects wore one-layer cotton garments during the driving simulation sessions. A specific study with garments having different types of fabric and a different number of layers could bring some insight into the limitations a cECG system can face in real-life use cases like working.

A driving simulator was used instead of an on-road approach to isolate driving movements from other types of noise-inducing sources like car vibration and road conditions. Those types of noise-inducing sources go beyond the scope of the present paper and could be separately investigated in another study.

In our study, to measure RR durations, we compared the FFT-based method with another method based on the Pan–Tompkins algorithm. The FFT approach was chosen for its robustness to noise, which is highly relevant in our application. Pan–Tompkins, while a classic method with known limitations, remains a widely recognized benchmark in the literature (for its high baseline accuracy, computational simplicity, and widespread implementation) and was therefore included as a reference point to contextualize the performance of our method. We acknowledge that there exist numerous other modern algorithms (wavelet-based methods, deep learning-based methods, etc.) [80,81,82,83,84]. However, a direct comparison with all of these methods was outside the scope of the present work.

The SQI is not without limitations. First, some types of noise have an overlapping spectrum with the signal of interest—notably motion artifacts and triboelectric effects, and this may impact the accuracy of the SQI as a signal quality metric. Since both motion artifact and triboelectric effects are transient, we mitigated the issue by calculating the SQI on 15-s sections of the signal and not the whole recording. We also capped the cECG absolute amplitude to a z-score of eight to eliminate high amplitude spikes, mostly related to motion artifacts. In the end, the SQI proved to be reliable since it accurately predicted RR duration accuracy, with the lowest RR duration accuracies being in the lowest range of SQI values.

### 4.4. Conclusions

In conclusion, this study demonstrated that a capacitive coupling sensor with textile electrodes can provide accurate heart rate monitoring, for a wide variety of designs and placements, despite challenging signal quality, and despite movements from legs and arms interacting with pedals and the steering wheel. Capacitive coupling sensors with textile electrodes are therefore suitable candidates for assessing the feasibility of using heart rate for applications such as alertness detection on the road.

## Figures and Tables

**Table 1:** Selection of subjects.

Subject (#)	Bustline (cm)	Waistline (cm)	Shoulder Line (cm)	Torso Length (cm)	Height (cm)
1	95	92	112	40	182
2	134	127	139	52	190
3	98	98	129	39.5	185
4	88	78	104	38	160
5	91	92	110	41	187.5
6	93	83	110	38	179
7	101	100	122.5	39	179

**Table 2:** Driving circuit segmentation. Table and caption reproduced from [60], distributed under the terms and conditions of the Creative Commons Attribution (CC BY) license.

**Segment (#)**	**1**	**2**	**3**	**4**	**5**	**6**	**7**	**8**	**9**	**10**
Radius (m)	2000	2000	400	400	850	400	400	850	400	400
Arc (m)	1531	1531	306	306	1202	306	306	1202	306	306
Direction	Right	Left	Right	Left	Left	Right	Left	Left	Right	Left
**Segment (#)**	**11**	**12**	**13**	**14**	**15**	**16**	**17**	**18**	**19**	**20**
Radius (m)	2000	2000	400	400	850	400	400	850	400	400
Arc (m)	1531	1531	306	306	1202	306	306	1202	306	306
Direction	Right	Left	Right	Left	Left	Right	Left	Left	Right	Left

**Table 3:** Sessions of acquisition. The subject performed the complete circuit 10 times, once for each acquisition session, assessing various electrode parameters, including height, spacing, angle, shape, area, and fabric type.

Session (#)	Height (cm)	Spacing (cm)	Angle (°)	Shape	Area (cm^2^)	Fabric	Electrode
1	15	16	0	S	26	E	A.73.50
2	25	16	0	S	26	E	A.73.50
3	35	16	0	S	26	E	A.73.50
4	25	12	0	S	26	E	A.73.50
5	25	20	0	S	26	E	A.73.50
6	25	16	0	S	58	W	A.73.7
7	25	16	0	S	58	E	A.73.41
8	25	16	0	R	58	E	A.73.42
9	25	16	90	R	58	E	A.73.42
10	25	16	45	R	58	E	A.73.42

**Table 4:** Effect of electrodes’ placements and design on signal quality.

Comparison #	Independent Variable	cECG Groups	Acquisition Session #
I	Height	15 cm	1
		25 cm	2
		35 cm	3
II	Spacing	12 cm	4
		16 cm	2
		20 cm	5
III	Angle	0°	8
		45°	10
		90°	9
IV	Shape	Square	7
		Rectangle at 0°	8
		Rectangle at 45°	10
		Rectangle at 90°	9
V	Size	26 cm^2^	2
		58 cm^2^	7
VI	Fabric	Woven	6
		Embroidered	7

**Table 5:** Signal quality index: rECG vs. cECG. The number of samples N is the number of cECG or rECG signals in the study.

	rECG SQI	cECG SQI
Median	4.29	0.69
Mean	4.4	0.78
STD	1.41	0.36
Range	4.97	1.53
N	70	70
*p* value	1.7 × 10^−21^	

**Table 6:** Error coverage rates for textile electrodes with the FFT-based method. The number of samples N is the number of cECG signals in the study.

	Percentage of Data Within ERP Range				
	[0, 5]	]5, 10]	]10, 15]	]15, 20]	]20, ∞[
Median	86	11	0	0	0
Mean	81	12	2	1	4
STD	18	8	4	3	10
Range	91	34	23	20	49
N	70	70	70	70	70

**Table 7:** Effect of electrodes’ height on signal quality. The number of samples N is the number of 15-s sections of signal: 245 sections = 7 subjects × 1 signal per subject × 35 sections per signal.

	Height: 15 cm SQI	Height: 25 cm SQI	Height: 35 cm SQI
Median	0.65	0.67	0.80
Mean	0.61	0.72	0.88
STD	0.28	0.37	0.53
Range	1.33	1.92	2.87
N	245	245	245
*p* value	15 cm vs. 25 cm: *p* = 4.8 × 10^−3^ 15 cm vs. 35 cm: *p* = 1.2 × 10^−4^ 25 cm vs. 35 cm: *p* = 4.4 × 10^−1^		

**Table 8:** Effect of width between electrodes on signal quality. The number of samples N is the number of 15-s sections of signal: 245 sections = 7 subjects × 1 signal per subject × 35 sections per signal.

	Width: 12 cm SQI	Width: 16 cm SQI	Width: 20 cm SQI
Median	0.94	0.67	1.20
Mean	0.96	0.72	1.21
STD	0.41	0.37	0.58
Range	2.11	1.92	2.82
N	245	245	245
*p* value	12 cm vs. 16 cm: *p* = 2.5 × 10^−7^ 12 cm vs. 20 cm: *p* = 6.9 × 10^−5^ 16 cm vs. 20 cm: *p* = 5.2 × 10^−17^		

**Table 9:** Effect of electrodes’ angle on signal quality. The number of samples N is the number of 15-s sections of signal: 245 sections = 7 subjects × 1 signal per subject × 35 sections per signal.

	Angle: 0° SQI	Angle: 45° SQI	Angle: 90° SQI
Median	0.58	0.65	0.69
Mean	0.61	0.65	0.76
STD	0.40	0.39	0.47
Range	2.82	1.75	2.67
N	245	245	245
*p* value	0° vs. 45°: *p* = 5.2 × 10^−1^ 0° vs. 90°: *p* = 8.6 × 10^−4^ 45° vs. 90°: *p* = 3.2 × 10^−3^		

**Table 10:** Effect of electrodes’ shape on signal quality. The number of samples N is the number of 15-s sections of signal: 245 sections = 7 subjects × 1 signal per subject × 35 sections per signal.

	Square SQI	Rectangle at 0° SQI	Rectangle at 45° SQI	Rectangle at 90° SQI
Median	0.70	0.58	0.65	0.69
Mean	0.73	0.61	0.65	0.76
STD	0.43	0.40	0.39	0.47
Range	2.07	2.82	1.75	2.67
N	245	245	245	245
*p* value	Square vs. Rectangle at 0°: *p* = 9.6 × 10^−2^ Square vs. Rectangle at 45°: *p* = 1.4 × 10^−3^ Square vs. Rectangle at 90°: *p* = 7.0 × 10^−1^			

**Table 11:** Effect of electrodes’ size on signal quality. The number of samples N is the number of 15-s sections of signal: 245 sections = 7 subjects × 1 signal per subject × 35 sections per signal.

	Area: 26 cm^2^ SQI	Area: 58 cm^2^ SQI
Median	0.67	0.70
Mean	0.72	0.73
STD	0.37	0.43
Range	1.92	2.07
N	245	245
*p* value	3.7 × 10^−1^	

**Table 12:** Effect of electrodes’ fabric on signal quality. The number of samples N is the number of 15-s sections of signal: 245 sections = 7 subjects × 1 signal per subject × 35 sections per signal.

	Fabric: Woven SQI	Fabric: Embroidered SQI
Median	0.66	0.70
Mean	0.64	0.73
STD	0.37	0.43
Range	1.78	2.07
N	245	245
*p* value	2.1 × 10^−3^	

**Figure A1:**
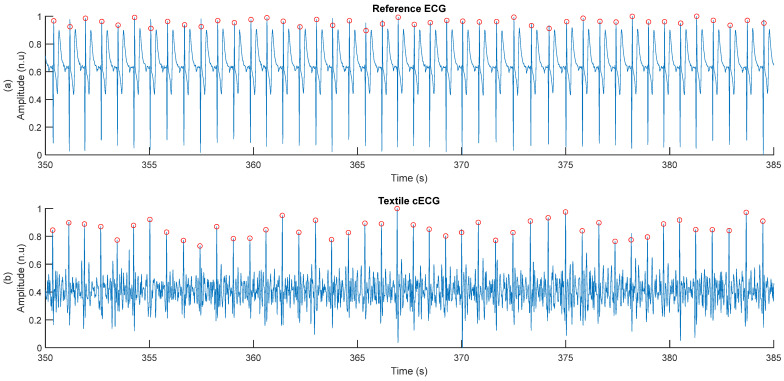
Detection of R peaks on 35-s strips of reference ECG and textile cECG. (**a**) Raw reference ECG. (**b**) Textile cECG filtered with high-pass (5th order Butterworth, 5Hz cutoff frequency) and low-pass (15th order FIR, 50Hz cutoff frequency) filters. Signals were taken from subject #5 in session #3. Amplitudes are in normalized units (between zero and one).

**Figure A2:**
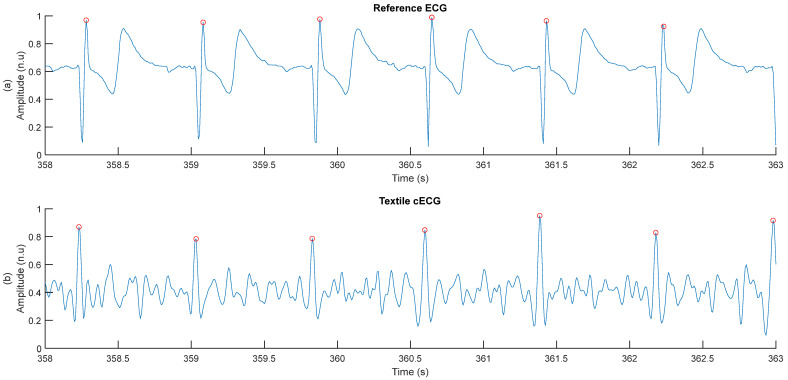
Close-up of Figure A1 over 5 s. (**a**) Raw reference ECG. (**b**) Textile cECG filtered with high-pass (5th order Butterworth, 5Hz cutoff frequency) and low-pass (15th order FIR, 50Hz cutoff frequency) filters. Signals were taken from subject #5 in session #3. Amplitudes are in normalized units (between zero and one).

**Figure A3:**
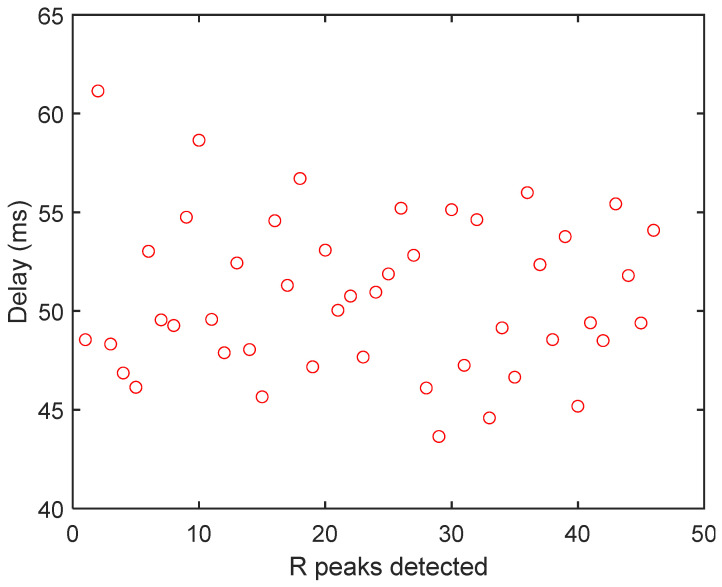
Scatter plot of delays between R peaks timing on reference ECG and R peaks timing on textile cECG.

**Figure A4:**
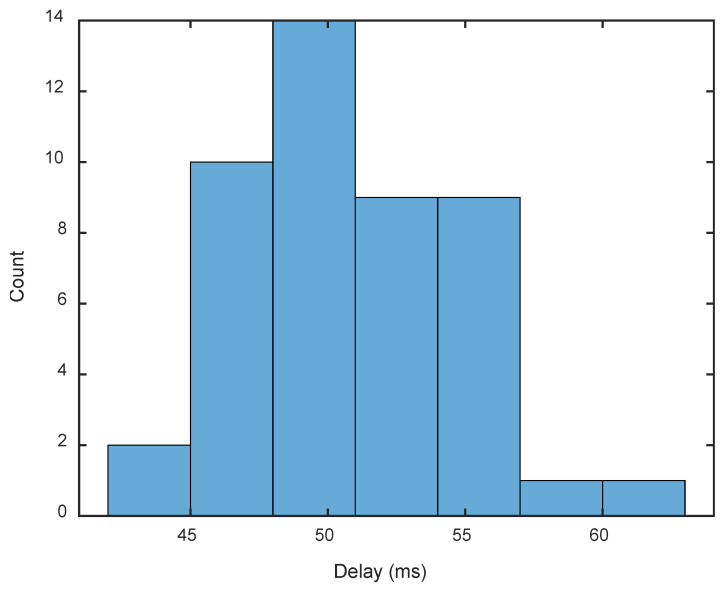
Distribution of delays between R peaks timing on reference ECG and R peaks timing on textile cECG.

## Data Availability

The data for the project were collected at the electrical engineering department, École de Technologie Supérieure. For ethical and confidentiality reasons, the authors cannot provide public access to them. Nevertheless, the authors agree to make data and materials supporting the results or analyses available for the investigation of scientific integrity if necessary.

## Appendix A

The performance of the Lab Streaming Layer synchronization software was tested by comparing the timing of R peak detections between reference and textile electrodes. We selected a 35-s strip of reference ECG and the corresponding textile cECG signals from subject #5 in session #3 (Figure A1). The criterium of selection was the ability to detect R peaks with the Pan–Tomkins algorithm on both signals. A five-second close-up of the signals (Figure A2) clearly shows a non-zero delay between R peaks timing on reference ECG and R peaks timing on textile cECG. As a matter of fact, there is a positive delay of around 50 ms, as shown in the scatter plot of Figure A3 and distribution of Figure A4. More precisely the set of delays had a median of 50 ms, an average of 51 ms, a standard deviation of 4 ms, and a range of 17 ms. The number of samples was 46.
